# Assessing the Single and Combined Toxicity of Chlorantraniliprole and *Bacillus thuringiensis* (GO33A) against Four Selected Strains of *Plutella xylostella* (Lepidoptera: Plutellidae), and a Gene Expression Analysis

**DOI:** 10.3390/toxins13030227

**Published:** 2021-03-22

**Authors:** Muhammad Zeeshan Shabbir, Ling He, Changlong Shu, Fei Yin, Jie Zhang, Zhen-Yu Li

**Affiliations:** 1 Institute of Plant Protection, Guangdong Academy of Agricultural Sciences, Guangzhou 510640, China; zeeshan@gdppri.com (M.Z.S.); linghe02@hotmail.com (L.H.); yinfei@gdaas.cn (F.Y.); 2 Guangdong Provincial Key Laboratory of High Technology for Plant Protection, Guangzhou 510640, China; 3 State Key Laboratory for Biology of Plant Diseases and Insect Pests, Institute of Plant Protection, Chinese Academy of Agricultural Sciences, Beijing 100094, China; clshu@ippcaas.cn (C.S.); jiezhang@caas.net.cn (J.Z.)

**Keywords:** *Bacillus thuringiensis*, biochemical assay, chlorantraniliprole, joint action toxicity, resistant management

## Abstract

Concerns about resistance development to conventional insecticides in diamondback moth (DBM) *Plutella xylostella* (L.)*,* the most destructive pest of *Brassica* vegetables, have stimulated interest in alternative pest management strategies. The toxicity of *Bacillus thuringiensis* subsp. *aizawai* (Bt GO33A) combined with chlorantraniliprole (Chl) has not been documented. Here, we examined single and combined toxicity of chlorantraniliprole and Bt to assess the levels of resistance in four DBM strains. Additionally, enzyme activities were tested in field-original highly resistant (FOH-DBM), Bt-resistant (Bt-DBM), chlorantraniliprole-resistant (CL-DBM), and Bt + chlorantraniliprole-resistant (BtC-DBM) strains. The Bt product had the highest toxicity to all four DBM strains followed by the mixture of insecticides (Bt + Chl) and chlorantraniliprole. Synergism between Bt and chlorantraniliprole was observed; the combination of Bt + (Bt + Chl) (1:1, LC_50_:LC_50_) was the most toxic, showing a synergistic effect against all four DBM strains with a poison ratio of 1.35, 1.29, 1.27, and 1.25. Glutathione *S*-transferase (GST) and carboxyl-esterase (CarE) activities showed positive correlations with chlorantraniliprole resistance, but no correlation was observed with resistance to Bt and Bt + Chl insecticides. Expression of genes coding for PxGST, CarE, AChE, and MFO using qRT-PCR showed that the PxGST and MFO were significantly overexpressed in Bt-DBM. However, AChE and CarE showed no difference in the four DBM strains. Mixtures of Bt with chlorantraniliprole exhibited synergistic effects and may aid the design of new combinations of pesticides to delay resistance in DBM strains substantially.

## 1. Introduction

The diamondback moth (DBM), *Plutella xylostella* (L.) (Lepidoptera: Plutellidae), is a major pest of *Brassica* vegetables, reported in more than 80 countries, and causes severe damage to vegetable crops in China and wherever it occurs [1,2,3,4,5,6]. The annual yield losses and cost associated with the management of diamondback moth are estimated to be billions of dollars worldwide [5]. Insecticides remain the primary approach for management of *P. xylostella*. The most commonly used insecticides for cruciferous vegetables in China are diamides, pyrethrins, avermectins, and *Bacillus thuringiensis* (Bt) [7]. Furthermore, the evolution of resistance to a wide range of insecticides, and subsequent spray failures, have been reported for *P. xylostella*, including Bt products [1,4,8,9].

The understanding of the resistance mechanism in target pests to insecticides is essential for an effective control strategy. Mechanism of resistance can involve higher levels of detoxifying enzymes or metabolic resistance, reduced penetration and changes in the target sites due to mutations [10,11,12]. The common mechanisms of metabolic resistance to insecticides include enhanced carboxyl-esterase (CarE), glutathione *S*-transferases (GST), and cytochrome P450 monooxygenase (MFO) activity [13,14,15]. The insensitivity of the target site to organophosphates and pyrethroids is related to the activity of acetylcholinesterase (AChE) [16]. The correlations between detoxifying enzymes and insecticide resistance have been reported extensively [4], including the overexpression of enzymatic detoxification involved in resistance in *P. xylostella* [9].

Transgenic crops expressing various Bt toxins have been extensively adopted to combat lepidopteran pests [17,18,19]. However, the widespread usage of a single transgenic product raises the prospect of insect resistance to Bt proteins, thus reducing the efficacy of Bt crops. Several lepidopteran pests have developed resistance to various Bt toxins, including Cry1Ac, Cry1Ie, Cry1F, and Cry1Ah, both in the field and in laboratory experiments [19,20,21,22]. To overcome this issue, Bt toxins are often used in combinations with other insecticides so as to delay resistance evolution in lepidopteran pests [23,24].

In the last decade, considerable progress has been made in the assessment of toxicity of combinations of toxins or mixtures against several lepidopteran pests. It is common to perceive that increased toxicity comes from exposure to compounds in a mixture or combination rather than to a single product. Chemical combinations can demonstrate additive, synergistic and antagonistic effects even if used in low concentrations [25]. Mixtures of numerous pesticides are being used against pests to help ensure crop production [26,27,28]. The strategy of using a mixture of chemicals has been studied extensively [23,29,30]. However, research on testing combinations of Bt toxins and insecticides is limited. Thus, determining how a mixture of Bt and pesticides affect DBM populations will throw much-needed light on how to improve the management of this major pest.

Successful development of resistance management strategies needs careful selection of the insecticide type to apply. Here, we assess whether a Bt product and chlorantraniliprole (Chl) will delay resistance evolution in strains of *P. xylostella*. Bt and chlorantraniliprole were chosen because no one has reported on their effects as a mixture/combination, and we do so using four strains of DBM. Here, we determined the detoxifying enzyme activity of glutathione S-transferase (GST), cytochrome P450 monooxygenase (MFO), acetyl-cholinesterase (AChE), and carboxyl-esterase (CarE) to find a correlation between the Bt product and chlorantraniliprole. In addition, we compared the gene expression among the DBM strains, using qRT-PCR. This study provides important information for developing successful resistance management strategies for DBM. It will help delay resistance evolution through the application of tactics such as Bt and chlorantraniliprole mixtures and rotation of insecticides with different modes of action.

## 2. Results

### 2.1. The Resistance Level of Greenhouse Strains of P. xylostella to Three Insecticides

Leaf-dip bioassays revealed that the different insecticides varied in their toxicities to the four DBM strains (Table 1). The chlorantraniliprole (CL)-DBM strain developed a moderate level of resistance (31.2-fold) to chlorantraniliprole compared with larvae from the field-original highly resistant (FOH)-DBM. Similarly, the Bt-DBM and Bt + chlorantraniliprole (BtC)-DBM strains showed a moderate level of resistance to chlorantraniliprole, with a resistance ratio of 24.2- and 27.3-fold, respectively (Table 1).

The Bt product (GO33A) displayed high toxicity to all four strains of DBM. The LC_50_ value of Bt-DBM was 16.29 mg L^−1^, showing an 18.7-fold resistance level compared to FOH-DBM. However, the BtC-DBM and CL-DBM showed little difference in their resistance to the Bt toxin. These data indicated that no cross-resistance was observed in these strains concerning Bt product (Table 1).

The LC_50_ of the binary mixtures of the Bt product and chlorantraniliprole (Bt + Chl) were determined to understand the interaction effect of these chemicals towards four strains of DBM. The strain selected with the Bt product (Bt-DBM) had a 6.5-fold resistance to the Bt + Chl mixture, and selection with chlorantraniliprole (CL-DBM) and BtC-DBM caused the same level of resistance to the binary mixture (Bt + Chl) (Table 1). No significant difference was observed in the LC_50_ values of the CL-DBM and BtC-DBM strains. Based on these results, the toxicity of the three tested insecticides to four greenhouse DBM strains was ranked as Bt > Bt + Chl > chlorantraniliprole.

### 2.2. Joint Action of Combinations

The combinations of the chemicals Bt and chlorantraniliprole against the four DBM strains showed synergistic, additive, and antagonistic interactions (Table 2). The combination of Bt + (Bt + Chl) exhibited the most synergistic effect with a poison ratio of 1.35 and was a major contributor to toxicity in the four DBM strains. However, the Bt + Chl (1:1) mixture exhibited an additive effect within the strains, except for FOH-DBM, and showed significantly lower toxicity. The toxicities of the treatments differed among the four DBM strains (Table 2).

### 2.3. Detoxifying Enzyme Activities

**Figure 1:**
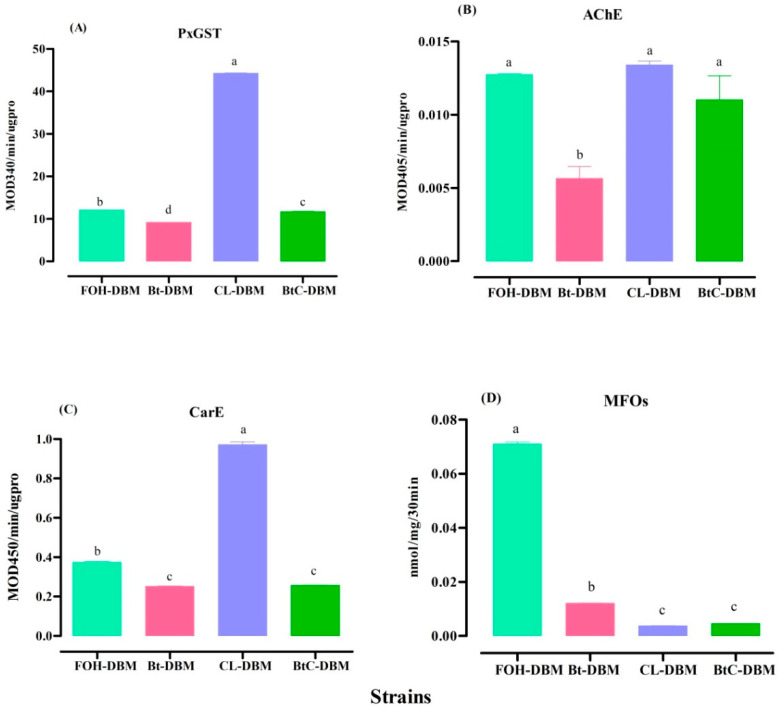
Activities of detoxifying enzymes in different strains of *Plutella xylostella*: (**A**) glutathione *S*-transferase (PxGST) activity; (**B**) acetylcholine esterase (AChE) activity; (**C**) carboxyl esterase (CarE) activity; (**D**) cytochrome P450 monooxygenase (MFO) activity. A significant difference was observed between the strains (df = 3, 11; PxGST: F =105624.61, *p* < 0.001; AChE: F = 14.37, *p* = 0.001; CarE: F = 2048.39, *p* < 0.001; MFO: F = 7292.5, *p* < 0.001). The bars labeled with the same letter indicate no significant difference while different letters are significantly different. Data represent the mean ± SE of three replications (GLM, LSD, *p* < 0.05).

The CL-DBM strain showed a significant increase in PxGST activity using CDNB as substrate, whereas the Bt-DBM and BtC-DBM strains revealed decreased PxGST activities compared to the FOH-DBM (Figure 1A). The increasing activity of PxGST might be contributing to resistance to chlorantraniliprole insecticide in the CL-DBM strain.

The acetyl cholinesterase (AChE) activity of the third instar larvae of the DBM strains indicated that only the Bt product significantly inhibits the AChE activity. Chlorantraniliprole and the mixture of Bt and chlorantraniliprole (Bt + Chl) showed an increased AChE activity but was not statistically significant compared to the FOH-DBM (Figure 1B).

The results of the carboxyl-esterase (CarE) activity indicated that CL-DBM showed significantly increased activity compared to the FOH-DBM, whereas the Bt-DBM and BtC-DBM strains showed considerably decreased CarE activity (Figure 1C).

The cytochrome P450 monooxygenases (MFO) activity was significantly enhanced in the FOH-DBM strain. However, the strains selected with Bt and chlorantraniliprole showed lower MFO activities (Figure 1D).

### 2.4. Expression Profiles of the Detoxification Genes

**Figure 2:**
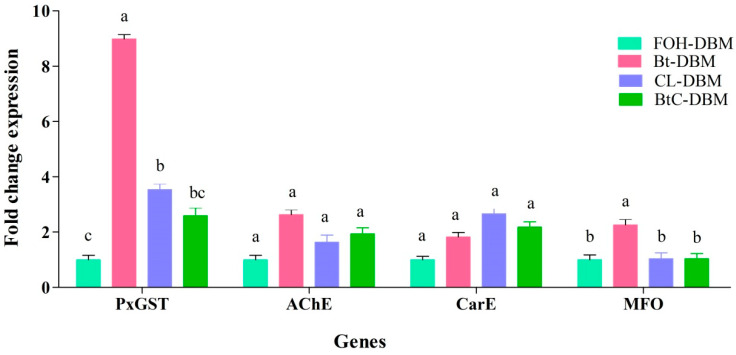
Differences in the fold-change expression level of the selected genes in four strains of *Plutella xylostella*. The gene expression data were generated by qRT-PCR analysis of four DBM strains. PxGST, glutathione *S*-transferase; AChE, acetylcholine esterase; CarE, carboxyl esterase; MFO, cytochrome P450 monooxygenases. The expression of the genes (PxGST and MFO) was significantly different between the strains (df = 3, 11; PxGST: F = 22.20, *p* = < 0.001; MFO: F = 9.49, *p* = 0.005). The bars labeled with the same letter are not significantly different and with different letter are significantly different (GLM, LSD, *p* < 0.05).

The expression levels of the four selected genes (PxGST, AChE, CarE, and MFO) in the four DBM strains differed. The expression level of PxGST was significantly higher in Bt-DBM (8.9-fold) followed by CL-DBM (3.5-fold) and BtC-DBM (2.6-fold) (Figure 2). Similarly, the gene expression of MFO was also significantly higher in Bt-DBM (2.62-fold) compared to the other strains. The expression of AChE and CarE was higher in the resistant strains compared to FOH-DBM but were not statistically significant (Figure 2).

## 3. Discussion

Chemical control is widely used for the management of lepidopteran pests, especially *P. xylostella*. However, insecticide resistance can significantly reduce the effectiveness of this strategy [31,32]. Pest resistance to pesticides fluctuates in members of the same populations due to the hereditary characteristics of strains [33], as well as many other factors [34]. Thus, the resistance evaluation of *P. xylostella* to commonly used pesticides is mainly related to developmental history, pesticide application frequency, and the actual selection pressure of each pesticide. Our study provides information that can help to extend the durability of Bt product to overcome resistance evolution based on an evaluation of four strains: three selected with Bt, chlorantraniliprole, and a mixture of Bt and chlorantraniliprole, and a control *P. xylostella* strain.

Among the insecticides tested, the Bt product was the most toxic against all four DBM strains, followed by the binary mixture of Bt and chlorantraniliprole (Bt + Chl) (Table 1). Chlorantraniliprole has shown less resistance when applied separately against the four DBM strains. The results show that BtC-DBM and Bt-DBM have the greatest LC_50_ values (188 and 211 mg L^−1^), resulting in a moderate cross-resistance to chlorantraniliprole with a resistance ratio of 24.2- and 27.3-fold, respectively. The resistance levels in these strains to chlorantraniliprole are not unexpected as *P. xylostella* can rapidly develop resistance to a wide range of insecticides [1]. However, the CL-DBM strain showed a 31.2-fold resistance compared to the FOH-DBM strain with respect to chlorantraniliprole. Clearly, the DBM strains exhibited different resistance levels to the tested chemicals. This variability in insecticide resistance of DBM has been explained by the difference in population and geographical distribution, as reported previously in numerous studies [7,35,36]. However, in our case, the strains originated from one location and were subjected to just two rounds of selection. Even with this limited selection, the toxicity of the three tested insecticides to the four DBM strains is ranked as Bt > Bt + Chl > chlorantraniliprole. A binary mixture of Bt and chlorantraniliprole did not show more toxicity to the four DBM strains compared to the Bt product when applied alone. These results are consistent with a previous study finding that the pyramids of the two toxins did not consistently delay resistance evolution relative to a single product in a lepidopteran pest [30].

The results of the joint action of insecticides indicated that the Bt product was more toxic to all four strains of DBM when mixed with the binary mixture Bt + Chl. In contrast, chlorantraniliprole showed low toxicity when mixed with the binary mixture Bt + Chl (Table 2). Our results suggest synergistic, additive, and antagonistic effects among the combinations of insecticides used against four strains of DBM. In particular, the mixture of Bt + (Bt + Chl) (1:1:1; LC_50_:LC_50_) produced a synergistic activity compared to other combinations in the field-original highly resistant strain. At the 50% effect level, synergism was confirmed for these mixtures in all four DBM strains. These results are consistent with a previous study, which indicated that *B. thuringiensis* and the insecticides binapacryl, chlordimeform, fentin hydroxide, and tricyclohexyltin hydroxide, in combination, were synergistic and controlled *P. xylostella* strains [37]. Similarly, these findings confirm a previous study that verified synergistic effects for a mixture of sulfonamides applied on *D. manga* [38], and reports have confirmed that mixtures of chemicals have higher toxicity to target pests [28,39]. However, the toxic effect of a binary combination of Bt + Chl (1:1; LC_50_:LC_50_) generated antagonistic effects with a poison ratio of 0.96 in FOH-DBM, but additive effects in all three selected strains of DBM. Combinations of insecticides play a meaningful role compared to single insecticide because they have multiple modes of action. Hence, chemical mixtures may be effective in management of lepidopteran pests; however, it is not clear how the synergism can be used positively. Moreover, a comprehensive understanding of the potential synergistic effects of the insecticides to non-target organisms must account for Bt and chlorantraniliprole combined.

The biochemical mechanisms of resistance have been used for studying toxin–invertebrate interactions directly in resistant and susceptible strains. It is speculated that detoxification enzymes, such as cytochrome P450 monooxygenases, glutathione *S*-transferase, acetyl-cholinesterase, and carboxyl-esterase, play an essential role in the metabolism of carbamates, pyrethroids, and novel insecticides in numerous insects, including *P. xylostella* [40,41]. We found biochemical resistance characteristics of Bt, chlorantraniliprole, and binary mixture of these chemicals against four DBM strains. Our enzyme assays showed that there was a high level of GST activity in CL-DBM compared to the other three strains. However, we found no association between GST activity and resistance to the Bt product and a mixture of Bt and chlorantraniliprole. This significant correlation between GST activity and chlorantraniliprole suggests that the GST gene probably confers resistance to this insecticide in *P. xylostella*. These findings are consistent with previous studies that the GST enzymes contribute to the detoxification of exogenous combinations of pesticides, such as indoxacarb, acephate, and chlorantraniliprole, in *P. xylostella* [42,43]. Furthermore, a positive correlation with GST activity was found in response to spinosad insecticide treatments [44,45,46]. The higher expression level of GST presumably raises the insecticide metabolism rates. Similarly, we found an association between the carboxyl-esterase (CarE) activity and resistance to chlorantraniliprole, in agreement with a previous study [34] that showed a correlation between CarE activity with abamectin resistance in *P. xylostella*. Previously, AChE and MFO have been found to be responsible for resistance to pyrethroids and other insecticides in *P. xylostella* [34,47]. However, we found no obvious correlation between MFO and AChE activities and resistance to Bt product and chlorantraniliprole.

Analysis of gene expression in four strains of DBM showed that PxGST displayed a significantly higher expression in the Bt-DBM strain than the other strains and the CarE gene expression was slightly reduced in the Bt product compared to chlorantraniliprole and the Bt + Chl mixture. Similarly, AChE, and MFO expression showed a moderate increase in the Bt-DBM strain compared to other resistant and FOH-DBM strains. Previously, a 1.29-fold higher ProPO activity level has been reported in a resistant *P. xylostella* strain compared to a susceptible strain [48]. The P450 and GST genes also have been identified to be involved in the detoxification of xenobiotics and conferring resistance [49,50]. In earlier work, it was reported that upregulation of genes played a significant role in the Bt toxin Cry1Ah resistance of *O. furnacalis* [51]. The higher expression of PxGST and other genes in the resistant strains as compared to the FOH-DBM strain suggested that these genes could have a significant role in Bt and chlorantraniliprole resistance of DBM. Moreover, resistance might be associated with the expression of multiple receptors between resistant and susceptible strains. Specifically, the higher expression of AChE to some extent in Bt-DBM could be due to specific binding/interference with neurotransmitters by a specific cry protein or combination of proteins. Follow-up work will be conducted to identify the specific genes linked to Bt resistance in DBM strains.

## 4. Conclusions

*Bacillus thuringiensis* subsp. *aizawai* (GO33A) had a considerable toxicity level, unlike the chlorantraniliprole insecticide and their mixture effect. The significant synergistic effects detected from binary mixtures of Bt + (Bt + Chl) and Chl + (Bt + Chl) (1:1; LC_50_:LC_50_) in all four different strains of *P. xylostella* suggests a practical approach that can delay or overcome common pesticide resistance mechanisms. It is remarkable that the mixture of these chemicals retained a high level of activity against four strains of DBM, where single product did not. A strong positive correlation between the enzyme activity, especially GST and chlorantraniliprole, shows the main detoxification mechanism of resistance. However, a correlation between enzyme activities and the Bt product was not substantially identified. These results support the idea of the potential use of the Bt product in ways that will considerably delay resistance in *P. xylostella*. Based on the evidence presented here, we conclude that the development of resistance in different strains of *P. xylostella* to chlorantraniliprole may be different depending on the concentrations of the applied pesticide. Furthermore, resistant strains, having developed metabolic resistance to certain tested pesticides, might have altered sensitivity to other pesticides with different modes of action. Finally, we cannot determine how mixtures of the toxin and insecticides will be applied in the future. However, these synergistic combinations may represent a first step towards the utilization of Bt products and insecticides that will considerably delay resistance in *P. xylostella*.

## 5. Materials and Methods

### 5.1. Selection of DBM Strains

The four strains in the present study were Bt-resistant (Bt-DBM), chlorantraniliprole-resistant (CL-DBM), Bt + chlorantraniliprole-resistant (BtC-DBM), and a field-original highly resistant (FOH-DBM) control. The latter was originally collected from the Shijing area, Guangzhou. Detailed information regarding FOH-DBM is provided in Table S1. This population was established in a greenhouse and reared on cabbage exposed to chlorantraniliprole insecticide, Bt product (GO33A), and their resistance level checked. The greenhouse was divided into four sections; in Section 1 no treatment was undertaken on plants infested with larvae; in Section 2, the cabbage plants were sprayed with Bt 4000X, 1.5 g Bt powder, and 6 L water; in Section 3, cabbage plants were treated with a mixture of Bt and chlorantraniliprole 2000X-1000X, 3 g Bt, plus 6 mL chlorantraniliprole into 6 L water; and in Section 4 plants were treated with chlorantraniliprole 300X, 20 mL 5% chlorantraniliprole, plus 6 L water. These four sections were subjected to a second treatment as follows: (1) no treatment applied (FOH-DBM strain); (2) sprayed with Bt 8000X, 1.25 g Bt powder GO33A, and 10 L water (the Bt-DBM strain); (3) treatment 16000X-3200X, 0.625 g Bt powder, 0.125 mL 5% chlorantraniliprole, and 10 L water (the BtC-DBM strain); and (4) treated with chlorantraniliprole 1600X, 6.25 mL 5% chlorantraniliprole, plus 10 L water (the CL-DBM strain) (Figure S1).

### 5.2. Insecticides and Chemical Reagents

The insecticides used were chlorantraniliprole (200 g L^−1^ SC), purchased from DuPont Agricultural Chemicals Ltd. (USA) and Bt product (GO33A), which was provided by Huazhong Agricultural University. The other chemicals and reagents were the same as described in our previous study [42].

### 5.3. Leaf Bioassay

The chlorantraniliprole, Bt product (GO33A), and the mixture of Bt and chlorantraniliprole (Bt + Chl) were used to determine the resistance level using leaf-dip bioassays. The resistance level was compared by determining the LC_50_ values of four DBM strains subjected to selection (see above). Insecticide solutions were prepared as serial dilutions with 100 mL distilled water containing 0.1% Triton X-100 (v/v). We used a control and 7 doses of each insecticide, 0.2–15 ppm (low-high) for the Bt product, 3.9–250 ppm for chlorantraniliprole, and 4–257 ppm for Bt + Chl (7.5 ppm of Bt + 125 ppm of chlorantraniliprole dissolved in 100 mL ddH_2_O), each dose with 3 replications for all DBM strains. For each concentration, three-leaf discs were dipped in each insecticide solution for 12–15 s, followed by air drying at room temperature for 2 h. The control leaf discs were dipped in a distilled water solution containing 0.1% Triton X-100. Treated leaf discs were placed individually into Petri dishes (8 cm D × 2 cm H). For each insecticide and dose treatment, ten 3rd instar larvae were placed on a treated cabbage leaf in a plastic petri dish and kept at 25 ± 2 °C, 65 ± 5% RH. Mortality data were assessed after 48 h. Larvae unable to move when touched with a fine-brush were considered to be dead. The control mortality was required to be <10% further to determine the medial lethal concentration (LC_50_) values.

### 5.4. The Joint Action of Insecticides

We assessed the effectiveness of combinations of Bt and chlorantraniliprole. We evaluated the joint action toxicity of the Bt and chlorantraniliprole mixtures against the four DBM strains. The constituents tested were (1) Bt + Chl; (2) Bt + (Bt + Chl); and (3) Chl + (Bt + Chl)—prepared based on their LC_50_ using the leaf-dip bioassay method as discussed above. The joint action of the Bt and chlorantraniliprole was assessed as described previously [39,52]. The poison ratios between the treatments were calculated according to the following:

Theoretical mortality of mixture M = Mortality of A × A% in M + Mortality of B × B% in M(1)

(2) $$\mathrm{Poison}\;\mathrm{ratio}\;\mathrm{of}\;a\;\mathrm{mixture}\;=\;\frac{\mathrm{Actual}\;\mathrm{mortality}}{\mathrm{Theoretical}\;\mathrm{mortality}}\;\times 100$$

In the equations, M denotes the mixture of A and B, and A and B are the tested insecticides. A poison ratio >1.0 indicates a synergistic effect, a poison ratio <1.0 an antagonistic effect, while a poison ratio equal to 1.0 is an additive effect.

### 5.5. Enzyme Activity Assay

To determine the glutathione transferase (GST), carboxyl-esterase (CarE), cytochrome P450 monooxygenases (MFO), and acetyl-cholinesterase (AChE) activity in the four selected strains of DBM, 30 3rd to 4th instar larvae were used for enzyme source preparation. Larvae were placed in a glass homogenizer filled with 1000 μL of 0.1 M sodium phosphate buffer (pH 7.0). After grinding, the homogenate was centrifuged at 12,000× *g* for 5 min at 4 °C. The resulting homogenate supernatant from each strain was diluted 10-fold and used as the enzyme source.

The GST enzyme activity was measured using 1-chloro-2,4-dinitrobenzene (CDNB) as a substrate [53]. Briefly, the working solution contained 100 µL of enzyme source, 50 µL of 1.2 mM CDNB, 50 µL of 0.2 M sodium phosphate buffer (pH 7.5), and 100 µL of 12 mM GSH. PBS was added in the control instead of the enzyme source. The optical density was recorded by using a Tecan Spark Spectrophotometer at 340 nm at an interval of 15 s for 5 min.

The CarE activity test was conducted by the method as reported in previous research [54]. Firstly, 50 μL enzyme sources of each strain were added separately into micro-plate wells followed 200 μL of 0.2 mol/L (pH 6.0) sodium phosphate buffer (prepared with 1 mmol L^−1^ α-NA acetate and 0.5 g L^−1^ fast blue salt). Optical density was recorded at 450 nm with intervals of 12 s for 30 times at 27 °C.

The AChE enzyme activity was determined by adopting the method as described previously [55], using iodide thiogenic acetylcholine (ATchI) as a substrate. The enzymes incubation was done by using a 50 µL enzyme source (50 µL contains 0.1% Triton-100), 0.1 mol/L phosphate buffer (pH 7.5, 100 µL ATchI), and 100 µL of 0.05 mmol/L DTNB placed in 96-well plate. The density was recorded at 405 nm with an interval of 30 s for 15 min.

For the P450 activity, p-NA was used as the substrate [47]. The total reaction mixture contained 56 μL of enzyme source homogenate, and 0.1 mol L^−1^ sodium phosphate solutions (pH 7.5) containing 1mM of DTT, 1 mM of PTU, 1 mM of EDTA, and 1 mM of PMSF. This solution was incubated in a water bath at 34 °C for 30 min. The absorbance at 405 nm was recorded using a micro-plate spectra photometer reader.

### 5.6. Validation of Gene Expression

The expression levels of four genes, namely, PxGST, AChE, CarE, and MFO, were investigated in four DBM strains. Total RNA was extracted from 3rd instar larvae of each DBM strain according to the manufacturer’s protocol of the EASYspin RNA isolation kit (Biomed, Beijing). Then, M-MLV reverse transcriptase (Takara Bio Inc, Kusatsu, Shiga, Japan) was used for the first-strand cDNA synthesis. The primers were synthesized by Invitrogen Trading (Shanghai) Co., Ltd. The primer details are provided in Table S2. The qRT-PCR (quantitative real-time PCR) was conducted according to the protocol of our previous research [51]. Actin was used as a housekeeping gene. The relative gene expression levels in four DBM strains were calculated using the 2^−ΔΔCT^ method.

### 5.7. Statistical Analysis

The LC_50_ with fiducial limits, chi-square (*x*^2−^) values, and the slope of the concentration–mortality and their standard errors were determined by probit analysis using POLO-PC [56]. All mortality (%) data were corrected using Abbott’s formula [57]. The data generated from enzyme analysis was analyzed by Microsoft Office Excel 2013 and shown as MOD min^−1^ µg protein^−1^ for PxGST, CarE, and AChE, and nmol mg protein^−1^ for MFO. Significant differences in the poison ratio, enzyme activities, and gene expression were analyzed by General Linear Model (GLM) followed by an LSD test (Statistix 9.1 version 2004). 

## Figures and Tables

**Table 1:** Toxicity of *Bacillus thuringiensis* (Bt) and chlorantraniliprole against four strains of *Plutella xylostella*.

Insecticide	Strains	Slope ± SE	LC_50_ (95% FL) (mg/L)	df	χ2	RR ^a^
Chlorantraniliprole	FOH-DBM ^c^	1.075 ± 0.277	7.76 (1.277–16.26)	5	0.744	-
	Bt-DBM ^d^	1.057 ± 0.209	188.08 (112.04–423.76)	5	1.642	24.2
	CL-DBM ^e^	0.902 ± 0.205	271.87 (143.82–927.94)	5	1.069	31.2
	BtC-DBM ^f^	0.791 ± 0.215	211.52 (104.06–901.522)	5	0.731	27.3
Bt	FOH-DBM	1.119 ± 0.175	0.87 (0.521–1.308)	5	0.686	-
	Bt-DBM	0.863 ± 0.166	16.29 (9.36–42.19)	5	0.627	18.7
	CL-DBM	1.726 ± 0.117	1.58 (1.13–2.18)	5	4.577	1.8
	BtC-DBM	1.592 ± 0.195	1.13 (0.7–1.69)	5	5.097	1.3
Bt + Chl ^b^	FOH-DBM	1.061 ± 0.253	5.71 (1.388–11.05)	5	2.251	-
	Bt-DBM	1.111 ± 0.219	36.99 (21.51–62.92)	5	0.694	6.5
	CL-DBM	1.022 ± 0.262	27.01 (16.39–42.98)	5	1.403	4.7
	BtC-DBM	1.134 ± 0.187	30.25 (18.96–47.21)	5	0.811	5.3

^a^ RR, resistance ratio = LC_50_ of a particular strain divided by LC_50_ of the field-original strain. ^b^ Bt + Chl, the insecticide mixture of Bt (G033A) and chlorantraniliprole (1:1). ^c^ FOH-DBM, the field-original highly resistant strain. ^d^ Bt-DBM, the resistant strain treated with Bt toxin (G033A). ^e^ CL-DBM, the resistant strain treated with chlorantraniliprole insecticide. ^f^ BtC-DBM, the resistant strain of DMB selected with a mixture of Bt and chlorantraniliprole.

**Table 2:** Joint action of Bt and chlorantraniliprole against *Plutella xylostella* strains.

Strains	Treatment ^a^	Actual Mortality (%) ^b^	Theoretical Mortality (%) ^c^	Poison Ratio ^d^
FOH-DBM	Bt + Chl	50.00 ± 5.77	51.67	0.96 ± 0.11 ^b^
	Bt + (Bt + Chl)	73.33 ± 3.33	53.33	1.35 ± 0.06 ^a^
	Chl + (Bt + Chl)	60.00 ± 3.33	48.33	1.24 ± 0.02 ^a, b^
Bt-DBM	Bt + Chl	56.67 ± 3.33	56.67	1.00 ± 0.05 ^b^
	Bt + (Bt + Chl)	76.67 ± 3.33	60.00	1.27 ± 0.10 ^a^
	Chl + (Bt + Chl)	66.67 ± 3.33	53.33	1.24 ± 0.06 ^a, b^
CL-DBM	Bt + Cl	53.33 ± 3.33	53.33	1.00 ± 0.06 ^b^
	Bt + (Bt + Chl)	73.33 ± 3.33	56.67	1.29 ± 0.10 ^a^
	Chl + (Bt + Chl)	63.33 ± 3.33	50.00	1.27 ± 0.06 ^a, b^
BtC-DBM	Bt + Chl	56. 67 ± 3.33	56.67	1.00 ± 0.06 ^b^
	Bt + (Bt + Chl)	73.33 ± 3.33	58. 33	1.25 ± 0.05 ^a^
	Chl + (Bt + Chl)	66.67 ± 3.33	55.00	1.21 ± 0.06 ^a, b^

^a^ Combination of Bt (G033A) and Chl (chlorantraniliprole) (1:1, LC_50_:LC_50_). ^b^ Actual mortality data are the mean of three replicates represented as the mean ± standard error (*n* = 3). ^c^ Mean values of the mortality of each insecticide in the mixture. ^d^ Poison ratio >1.0 defined as a synergistic effect, 1.0 as an additive effect, and <1.0 as an antagonistic effect. A significant difference was observed between the treatments (df = 2,8; FOH-DBM: F = 7.89, *p* = 0.0209; Bt-DBM: F = 6.72, *p* = 0.0294; CL-DBM: F = 6.70, *p* = 0.0295; BtC-DBM: F = 5.44, *p* = 0.0449). Data are the mean of three replications and represented as the mean ± standard error. Means in the same column followed by the same letter are not significantly different (GLM, LSD, *p* < 0.05).

## Data Availability

The data presented in this study are available in Supplementary Materials.

## Supplementary Materials

The following are available online at https://www.mdpi.com/2072-6651/13/3/227/s1, Figure S1: Experiment layout and treatments application in the greenhouse; Table S1: Resistance of FOH-DBMs to different insecticides, Table S2: Primer sequences and amplification characteristics of the candidate reference genes for qRT-PCR studies in Plutella xylostella.
