# Supplementary material for: Assessing the Single and Combined Toxicity of Chlorantraniliprole and Bacillus thuringiensis (GO33A) against Four Selected Strains of Plutella xylostella (Lepidoptera: Plutellidae), and a Gene Expression Analysis
Source: Toxins (Basel). 2021 Mar 22;13(3):227. doi: 10.3390/toxins13030227 (PMC8004223; doi:10.3390/toxins13030227)
Supplement: Supplementary file 1 [file toxins-13-00227-s001.pdf]

Supplementary Materials: Assessing the Single and Combined Toxicity of Chlorantraniliprole and *Bacillus thuringiensis* (GO33A) against Four Selected Strains of *Plutella xylostella* (Lepidoptera: Plutellidae), and a Gene Expression Analysis

Muhammad Zeeshan Shabbir, Ling He, Changlong Shu, Fei Yin, Jie Zhang and Zhen-Yu Li

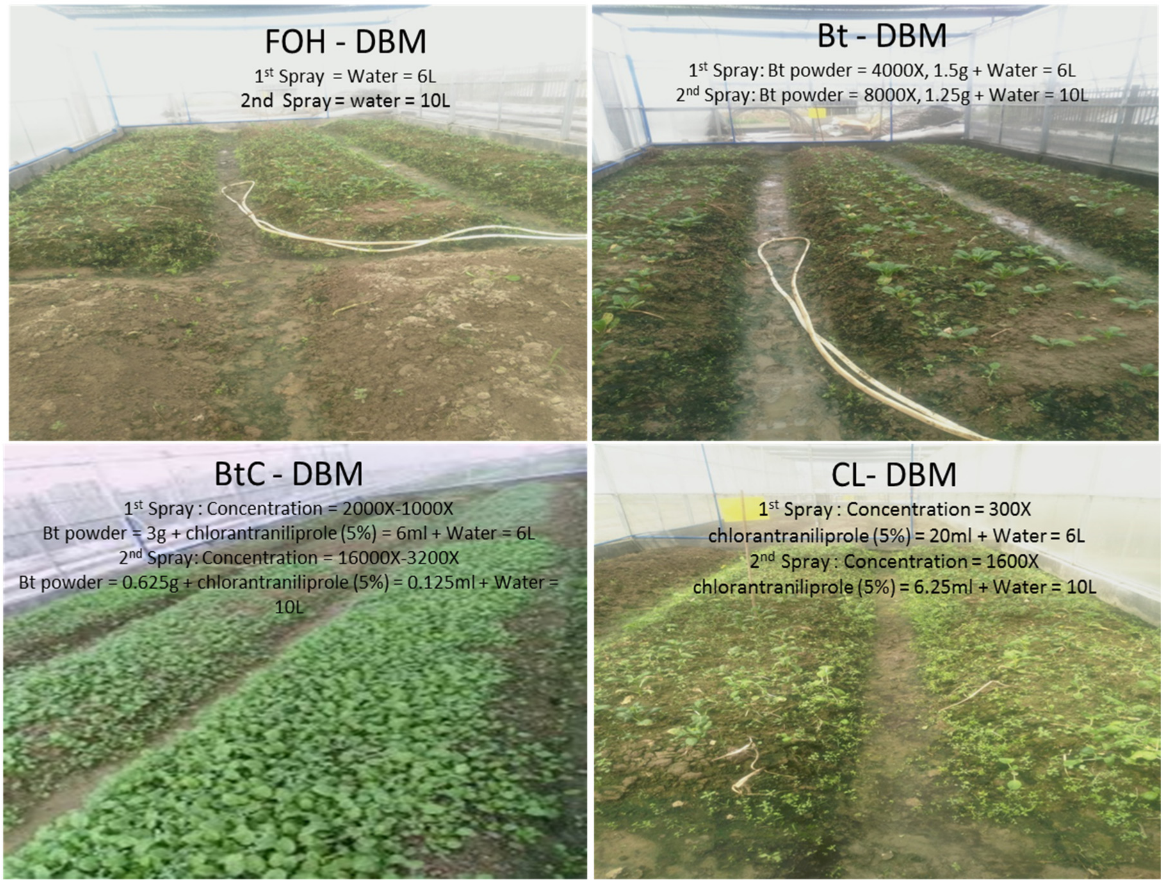

Figure S1. Experiment layout and treatments application in the greenhouse. Field-original highly resistant diamondback moths (FOH-DBMs) were originally collected from the Shijing area, Guangzhou. This population was established in the greenhouse. This population was established in the greenhouse and reared on cabbage exposed to Bt (GO33A), chlorantraniliprole and a mixture/combination of insecticides (Bt ± chlorantraniliprole). The greenhouse was divided into four sections. The experiment was carried out in a randomized complete block design (RCBD). The cabbage plants were sown in rows in each section. In total, there were 200 plants/row, with 30 cm row-to-row and 15 cm plant-to-plant distances. The total area of section A and B was 66.7 m^2, and for section C and D, it was 71.3 m^2. Cultural practices were adopted to maintain a good crop and rearing conditions were maintained at 26 ± 2 °C, 75 ± 5 RH, and a 12:12 (D:L) photoperiod. Two treatments were performed: the 1st treatment was applied on 31 October 2017 and the 2nd treatment was performed on 6 March 2018. The larval infestation (~15–20 larvae/replicate) was observed from 15 randomly selected plants from each section on a weekly basis.

Table S1. Resistance of FOH-DBMs to different insecticides.

Insecticide	Regression equation	LC50 (95% FL) (mg/L)
2% Fungus	Y = 1.238x − 0.645	3.319 (2.063–4.663)
19.2% Butyl ether urea	Y = 0.819x − 1.477	58.49 (24.42–96.49)
10.85% Bromopyron	Y = 1.25x − 2.162	53.693 (36.56–73.33)
6% Ethyl spinosad	Y = 0.896x + 0.171	0.644 (0.278–1.047)
5% Acetamiprid	Y = 0.868x − 1.923	163.799 (97.996–384.727)
5% Chlorantraniliprole	Y = 1.61x − 4.281	10.382 (6.271–17.499)
6% Bt	Y = 0.651x + 0.039	0.871 (0.362–1.618)
20% Chlorine	Y = 0.671x − 0.931	24.398 (11.45–42.94)
24% Cyanfludizone	Y = 1.245x − 1.758	25.831 (18.919–35.853)

Table S2. Primer sequences and amplification characteristics of the candidate reference genes for qRT-PCR studies in *Plutella xylostella*.

Gene	Primer sequences (F/R) (5′ – 3′)	Amplification Size (bp)
Glutathionse S-transferase	CTCTGTACCCCAAGGACGTG<br>TGTCCTTGAGCATCTTGTCG	150
Acetyl cholinesterase	TTTACGGCGATCCGTTCTTC<br>CGGGAACTTGGGCTTGAATA	200
Carboxylesterase	TCGGAAATGAGAAAGTCACACG<br>CCCAGGCAGAATGTGTTCTAA	200
Multi-function oxidase	GCAGTGAAGTTGGCTACGAC<br>GTAGTCCTCCACCACCTCAC	180
Actin	CAGGGAGTGATGGTCGGTAT<br>GACACGCAGCTCGTTGTAGA	168
